# Comparing the Efficacy of Mtwo and D-RaCe Retreatment Systems in Removing Residual Gutta-Percha and Sealer in the Root Canal

**Published:** 2012-08-01

**Authors:** Hengameh Akhavan, Yaser Khalil Azdadi, Shahram Azimi, Bahare Dadresanfar, Anahid Ahmadi

**Affiliations:** 1. Department of Endodontics, Dental Branch, Azad University of Medical Sciences, Tehran, Iran; 2. Dentist, Tehran, Iran

**Keywords:** D-Race, Gutta-Percha, Mtwo, Root Canal Sealer

## Abstract

**Introduction:**

Retreatment is performed in teeth with unsuccessful root canal therapy or persistent apical lesion. The most important factor for achieving successful retreatment is thorough cleaning and reshaping. NiTi files and rotary instruments are widely used for the removal of obturatants. This study compared the ability of Mtwo and D-RaCe retreatment systems to remove residual gutta-percha and sealer within the root canal after retreatment.

**Materials and Methods:**

This in vitro experiment was performed on sixty extracted human teeth. The samples were cut at the CEJ level, manually prepared, filled with gutta-percha and AH26 and finally stored at 37ºC for two weeks. Samples were then randomly divided into two groups. Group 1 was retreated with Mtwo and Group 2 with D-RaCe. Both groups were then divided into two subgroups retreated either with or without solvent. Teeth were then vertically sectioned for evaluation of residual filling materials on the canal walls. A microscopic assessment at 16× magnification was performed. T-test statistical analysis was used to compare the data.

**Results:**

Comparison between the Mtwo and D-RaCe rotary systems revealed no significant differences in residual gutta-percha or sealer on canal walls (P=0.2). The study revealed a negative effect of solvent on removal of gutta-percha and sealer in both the Mtwo and D-RaCe systems.

**Conclusion:**

Mtwo and D-RaCe retreatment files removed residual gutta-percha and sealer similarly; there was no significant difference between them.

## Introduction

Retreatment is indicated for the healing of periradicular lesions, in cases of unsuccessful root canal therapy and/or persistent apical lesion [1]. The crucial factor for achieving successful retreatment is thorough reshaping and cleaning of the canals to eliminate bacteria [1][2][3]. A success rate of 74-98% is reported for the nonsurgical retreatment procedure [4]. The increasing patient demands for saving teeth and the 10% possibility of root canal failure signifies the importance of the retreatment procedure [4][5].

Currently, removal of root canal filling materials is performed by means of solvents, hand and rotary instruments and ultrasonics [6]. NiTi files are also effective for quick canal preparation [7]. Foschi et al. demonstrated Mtwo files to be more efficient compared to ProTaper in canal cleaning. A study by Thomas revealed Race to be more effective than the ProTaper system, leaving less residual gutta-percha and sealer [7][8][9]. In Bramante’s study of Mtwo, ProTaper and hand instruments, none of the rotary systems was capable of complete material removal; however, ProTaper seemed to be faster in this regard, yet it released more heat.

Mtwo appeared to release less heat and had less effect on the removal of gutta-percha and sealer [10]. Cunba et al. reported that Resilon removal was more effectively carried out compared to AH Plus removal. The cleaning working time was not significantly different between the two groups.

A scanning electron microscope (SEM) study showed remaining material in the apical region [11]. Shirrmeister et al. concluded that canals retreated with Race had less gutta-percha and sealer debris compared to groups retreated with H-file, Flex master or ProTaper [12]. H-file and Flex master had the highest rates of residual gutta-percha, with a significant difference compared to the Race group. Schirrmeister et al. demonstrated that Epiphany could be more effectively removed when compared to gutta-percha, with hand instruments and the Race rotary system; however the difference was insignificant [13]. Oliveira et al. reported no significant difference in remaining filling materials between gutta-percha /AH plus and Resilon/Epiphany groups when Liberator files were used for retreatment [14]. K3 file was more efficient and faster both for gutta-percha/AH plus and Resilon/Epiphany groups. In the Resilon/Epiphany group, material was effectively removed by both K3 and liberator. Schirrmeister et al. demonstrated microscopic evaluation to be more efficient for the detection of remaining filling materials, especially gutta-percha [15]. In a study by Delboni et al., stainless steel hand instruments appeared to leave less filler compared to groups retreated with chloroform or chlorhexidine [16]. None of the mentioned studies had compared the effects of Mtwo and D-RaCe files on remnant gutta-percha and sealer in retreatment procedures [11][12][13][14][15][16][17].

The aim of this study was to compare the efficiencies of Mtwo and D-RaCe rotary systems on the amount of residual gutta-percha and sealer in root canal.

## Materials and Methods

Sixty extracted lower first molars were used in this experimental study. In order to completely remove soft tissue from the samples’ external surfaces, they were placed in 5.25% NaOCl for one hour and then rinsed and immersed in normal saline. The average root length was determined to be 16 mm. The teeth were decoronated to have similar lengths. Canals were then manually prepared with the step-back technique. The working length was determined with a #10 K-file (Maillefer, Dentsply, Swiss) which was inserted into the canal so that it could be observed from the apical foramen. The working length was then determined to be 1 mm shorter than the inserted file. Root curvature was determined by radiography (PA film, Kodak-France) with the #10 file and set at less than 20 degrees [18]. Canals were then filed to working length and irrigated with 5.25% NaOCl. Patency was performed with the #10 file followed by recapitulation after subsequent filing. Canals were flared up to file #60. EDTA (17%) was used for smear layer removal followed by canal irrigation with 5.25% NaOCl for the removal of irrigated organic material. Canals were finally irrigated with 10 mL distilled water, and dried with paper cones #35. Suitable gutta-percha was chosen according to the working length followed by a control radiograph. The selected cone was then soaked with AH26 and reinserted into the canal. A suitable spreader was inserted with a light force to within 2 mm of the working length to laterally pack the gutta-percha. Lateral cones were then soaked with the sealer and inserted into the canal until the canal was completely filled, the residual gutta-percha was removed with a hot excavator, and the canal was vertically packed with a condenser. Canal orifices were sealed with temporary material.

Two radiographs with 0.4 sec exposure time were then taken from a distance of 15 cm, both mesiodistally and buccolingually. Roots were then preserved at a temperature of 37ºC temperature for 2 weeks. The samples were finally randomly divided into two groups of 30 samples:

Group 1: Retreated with Mtwo, subsequently divided into Groups A and B, retreated either with or without the use of solvent, respectively.

Group 2: Retreated with D-RaCe, divided into Groups C and D, retreated either with or without the use of solvent, respectively.

In Groups A and B, 2 mm of canal filling material were removed with Gates Glidden #3. A chloroform drop was poured on the gutta-percha in Group A. An Mtwo file #20/50 at 280rpm with 120 gcm torque (Endo IT Professional engine) was used in the canal for 10 seconds to permit the file to enter the canal sufficiently. The canals were then irrigated with 5.25% NaOCl followed by a drop of chloroform. Gutta-percha removal proceeded with Mtwo file #15/05 with 30gcm torque for 10 seconds. Canals were again irrigated with NaOCl and checked with hand instrument #35. Mtwo #35 and 40 with 4% flaring was used to assure thorough cleaning. After subsequent filing, irrigation with NaOCl 5.25% was carried out throughout the whole process.

In Groups C and D, 2 mm of canal filling material were removed with Gates Glidden #3. Chloroform drop was released on the gutta-percha in Group C only. A D-RaCe file size DR1 at 1000rpm was used for 10 seconds in the canal with the crown-down technique. The canals were then irrigated with 5 mL of 5.25% NaOCl, followed by a chloroform drop and a 10 second use of DR2. For assurance of clearance, a #35 file was used. Both a #35 and #40 file with 4% flaring were utilised to complete the cleaning process.

A vertical groove was cut with diamond disks on the buccal and lingual side of each sample. Samples were then cut in half with a chisel. For further assessment, a 16× mag. stereomicroscope (Olympus, SZM9, Japan) was used on the apical (1 mm coronal of apex), middle (7 mm coronal of apex) and coronal (12 mm coronal of apex) portions of the samples. Images were then processed with AutoCAD 2009 software. For the comparison of remaining filling material in different sections of different groups, an independent sample t-test was used.

## Results

Due to the normal distribution of the data, an independent t-test statistical analysis was utilised to compare the mean percentages of remaining gutta-percha and sealer in each group. Mean percentages of remaining gutta-percha and sealer within each group for “with solvent” and “without solvent” were assessed separately; the t-test was used in each coronal, middle and apical section of Mtwo and D-RaCe Independent sample (Table 1). The analysis revealed no significant difference between Mtwo and D-RaCe when no solvent was used. To assess the effect of solvent on gutta-percha and sealer removal within each study group in the three sections, t-test was ustilised again (Table 2);.analysis revealed that solvent significantly decreased the removal of gutta-percha and sealer when using Mtwo or D-RaCe in the coronal and middle sections of the samples. However, in the apical region, the presence or absence of solvent had no effect on the removal of filling material when using the two studied systems.

**Table 1:** Mean percentage of remnant gutta-percha and sealer in different canal sections

		**X(SD)**	
**File**	**Root Segment**	Solvent	Solvent free
**Mtwo**	**Coronal**	51.9(7.1)	32.8(6.4)
	**Middle**	45.6(8.8)	31.8(7.5)
	**Apical**	41.3(6.8)	43.4(7.7)
**D-RaCe**	**Coronal**	48.7(7.5)	29.2(6.5)
	**Middle**	46.5(7.9)	32.1(6.0)
	**Apical**	43.9(4.4)	44.3(5.4)

**Table 2:** Comparison of Mtwo and D-RaCe instruments’ ability to remove gutta-percha/sealer +/- solvent

**Root segment**		**Coronal**	**Middle**	**Apical**
**Group**	**Solvent**			
**Mtwo**	**With**	32.8±6.4	31.8±7.5	43.4±7.7
	**Without**	51.9±7.1	45.6±8.8	41.3±6.8
**Result**		P<0.0001	P<0.0001	P<0.4
**D-RaCe**	**With**	29.2±6.5	32.1±6	44.3±5.4
	**Without**	48.7±7.5	46.5±7.9	43.9±4.4
**Result**		P<0.0001	P<0.0001	P<0.8

## Discussion

The current study focused on the ability of Mtwo and D-RaCe rotary systems on removing sealer and Gutta-percha after preparation and cleaning of the canal in retreatment cases. The results demonstrated both systems to be efficient. The use of solvent was shown to have a negative effect on removal of filling material with both the Mtwo and D-RaCe systems. The D-RaCe rotary system consists of 2 files (DR1-DR2) especially designed for retreatment. DR1 is a 15.8 mm in length, size 30 file with a cutting tip, 10% tapering and 1000 rpm, capable of 1/3 coronal cleaning. DR2 is a 25.16 mm in length, size 25 file with 4% tapering and 600 rpm set for 2/3 apical cleaning [19]. The Mtwo rotary system also has two specifically designed files with cutting tips for retreatment. 280rpm is recommended for this system. The highest torque is 30gcm for the 15.05 file and 25.5 gcm for the 120 file [20].

In the Mtwo file, the distance between cutting edges (pitch) is increased from the tip of the instrument to the handle. The depth of the space designed for dentine removal is increased behind the blades, which provides the largest space for dentine removal and leads to more efficient gutta-percha and sealer removal [21]. The Mtwo file has an H file-like motion (up and down). Its capacity for good material removal is due to its structure. The H file has a positive Rake angle, making dentine removal efficient; it comes with a #25 file with 0.05 tapering in the coronal and a #15 file with 0.05 tapering in the middle and apical section. The D-RaCe system consists of two files: DR1 (#30 with 10% flaring for coronal section) and DR2 (#25 with 0.04 flaring for middle and apical sections. The space for dentine removal on the back of the blades is deep, and provides sufficient space for the exit of dentinal debris, contributing to superior removal of filling material [19].

Use of solvent in the middle and coronal sections of both study groups led to gutta-percha and sealer residue on the canal walls. Solvent might have softened the gutta-percha and modified its structure to a viscous and highly-adhesive material. Considering the resemblance of the Mtwo file structure to that of H files, it is reasonable to conclude that Mtwo, like the H file, tends to remove filling material in a Bulk form; hence the aforementioned alteration in the structure of gutta-percha could make it difficult to be removed. Chloroform is a Class 2B carcinogenic material and is toxic when adjacent to live tissues [22]. The current results showed no greater efficiency of material removal when using this solvent. It could therefore be concluded that its use in retreatment with either the Mtwo or D-RaCe rotary systems might not be necessary. In the current study, AH 26, an Epoxy resin, was used.

The desirable characteristics of the material, such as good flow ability, adhesion to dentinal walls and sufficient working time, have made it a good choice for root canal treatment [4]. Many studies have shown NiTi files to be safe when removing gutta-percha and sealer [20]. Posterior teeth are more prone to caries and root canal therapy, and, consequently retreatment. In this study, a distal root of the mandibular first molar was used due to its less curved canals. Sample selection was done regarding canal curves (less than the 20 degrees mentioned by Shneider) [23]. This is an advantage of this study as canal curves were not considered in previous studies.

Initial canal preparation was done by means of hand instruments. Fridman and Moshonov suggested that an increase in canal size decreased the remaining material [20][21][22][23][24][25]. Gates Glidden was used for removal of filling materials from the orifices, which made solvent placement and penetration easy and practical [12][14][22][24]. The larger sample group in the current study compared to similar previous studies, and its quantitative assessment, increases its accuracy [20][21][22][24][26]. Both the Mtwo and D-RaCe rotary system were efficient in canal cleaning.

Although radiographic evaluation showed no remnant gutta-percha in any of the study groups, no sample was clean of filling materials upon microscopic evaluation; this highlights the need for irrigation materials and cleaning in the retreatment process.

## Conclusions

The amount of gutta-percha and sealer remaining after retreating with Mtwo and D-RaCe was not significantly different; both systems were capable of efficient material removal. The use of chloroform solvent with both Mtwo and D-RaCe systems had a negative effect on the elimination of filling material. Its use is therefore not recommended.
